# Supplementary material for: Not simply a matter of parents—Infants’ sleep-wake patterns are associated with their regularity of eating
Source: PLoS One. 2023 Oct 5;18(10):e0291441. doi: 10.1371/journal.pone.0291441 (PMC10553286; doi:10.1371/journal.pone.0291441)
Supplement: S1 Protocol — (DOCX) [file pone.0291441.s001.docx]

## S1 Protocol. DNA extraction from stool and processing of gut microbiota

Gut microbiota profiles were computed based on stool samples collected by the parents at each assessment time point. Samples were stored at approx. 4°C (fridge temperature) at the families’ home, transported within 96 hours to the laboratory under consistent temperature], separated into 200 mg aliquots, and stored at -50 °C until further processing. For DNA analysis, total DNA was extracted using a PowerSoil DNA Isolation Kit (MOBIO Laboratories, Carlsbad, CA, USA), following the manufacturer’s instructions. To prepare 16S rRNA gene amplicon libraries we used primers targeting the V3 region^58^ with adapters for the Nextera Index Kit® (Illumina, CA, USA): NXt_338_F: 5′- TCG TCG GCA GCG TCA GAT GTG TAT AAG AGA CAG ACW CCT ACG GGW GGC AGC AG -3′ and NXt_518_R: 5′- GTC TCG TGG GCT CGG AGA TGT GTA TAA GAG ACA GAT TAC CGC GGC TGC TGG -3′. The steps to obtain the amplification profile, barcoding, amplicon library purification, and sequencing were realized as described previously^59^. The quality check was performed on the raw dataset (2x151bp, pair-ended) with FastQC^60^. The data was then trimmed for overlapping reverse and forward reads using SeqKit^61^. The reads were merged using FLASH (v1.2.11)^62^, with an overlap comprised between 15 and 300, and a maximum mismatch density of 0.25. To determine and trim primer regions Cutadapt (v1.12)^63^ was used with an error rate of up to 0.01. Prinseq^64^ was used for the quality filtering and removed reads when there were ambiguous nucleotides or when the mean quality was lower than 20. Zero-radius Operational Taxonomic Units (zOTUs) were determined through UNOISE (USEARCHv10.0.240)^65^ and taxonomy predictions were based on Greengenes^66^. OTUs served as the basis for computation of alpha diversity, bacterial maturation index, and enterotype.

To control for bias based on processing stool samples in batches, batch number was used as a control variable in all gut microbiota analysis. Samples containing reads below 50’000 reads were excluded (n=6). Remaining samples were rarefied to the lowest number of counts (50’268) in the remaining samples. Rarefaction led to 1430 amplicon sequencing variants. Autoclaved water samples exposed to the same workflow served as negative controls. They contained reads between 2 and 150’785, with zOTU2 (*Enterobacteriaceae*) being the most abundant in 30% of the negative controls. zOTU2 was present in 10% of the stool samples. The other zOTU detected in the negative controls were rarely present in the stool samples. zOTU2 did not have any significant influence on gut microbiota markers of this study (*i.e.*, gut microbial diversity, bacterial maturation index, enterotype). Detected bacterial taxa had to be present in at least 20% of samples across 3-, 6- and 12-months and represent at least 1% of the bacteria measured to be included in further analysis.
