# Supplementary figures and images for: Not simply a matter of parents—Infants’ sleep-wake patterns are associated with their regularity of eating
Source: PLoS One. 2023 Oct 5;18(10):e0291441. doi: 10.1371/journal.pone.0291441 (PMC10553286; doi:10.1371/journal.pone.0291441)

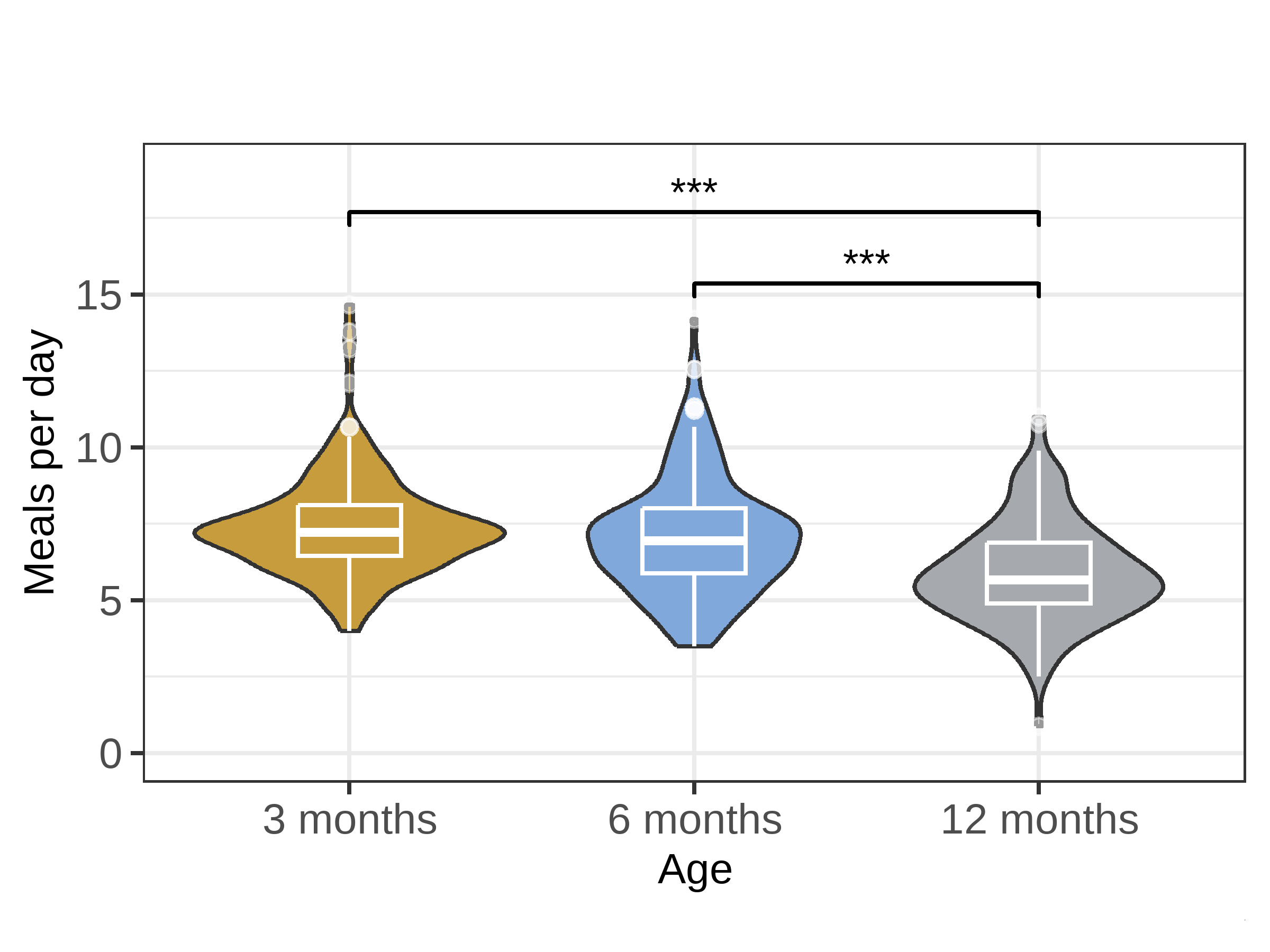

Supplement: S1 Fig — (TIF) [file pone.0291441.s002.tif]

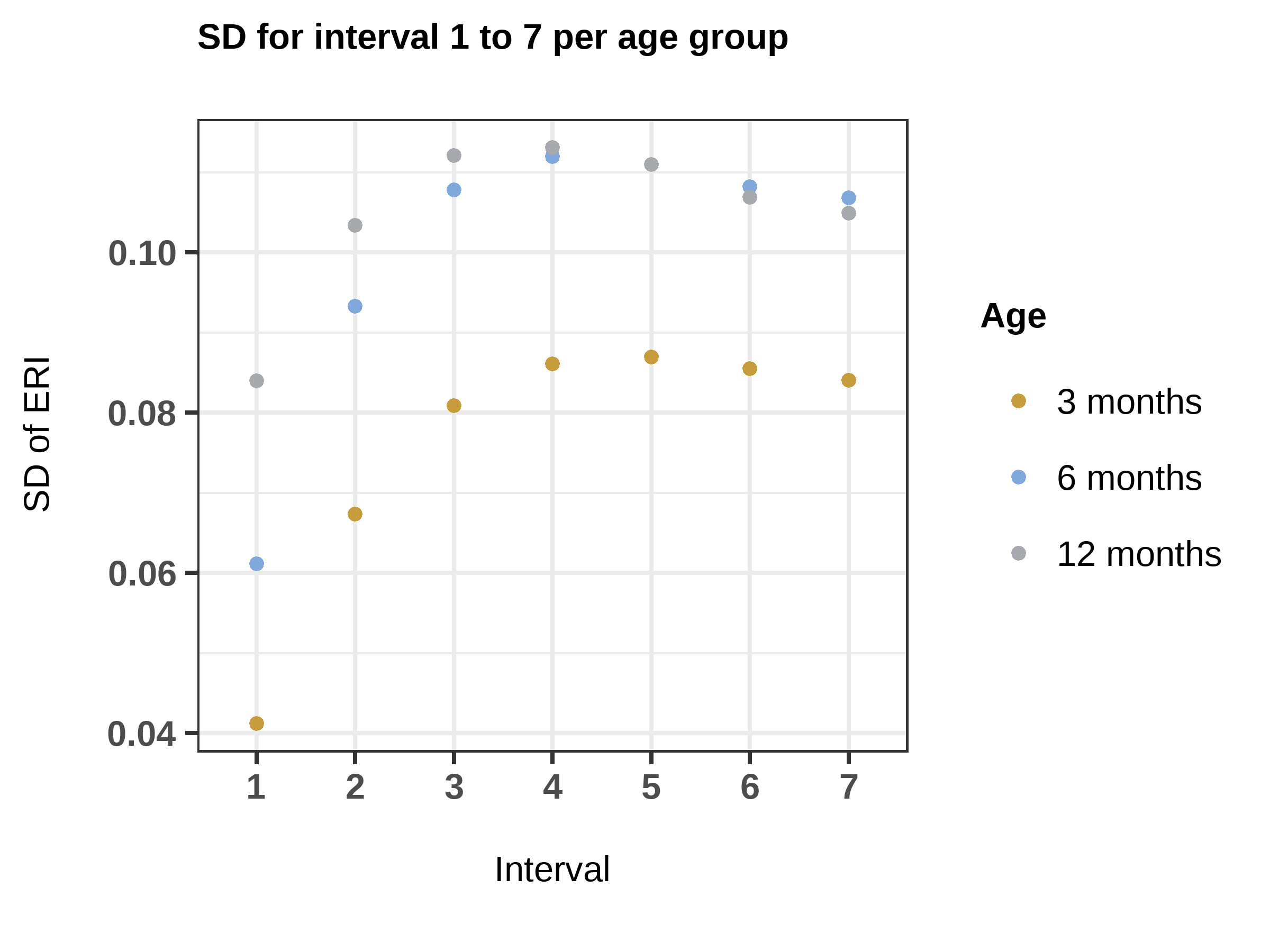

Supplement: S2 Fig — (TIF) [file pone.0291441.s003.tif]

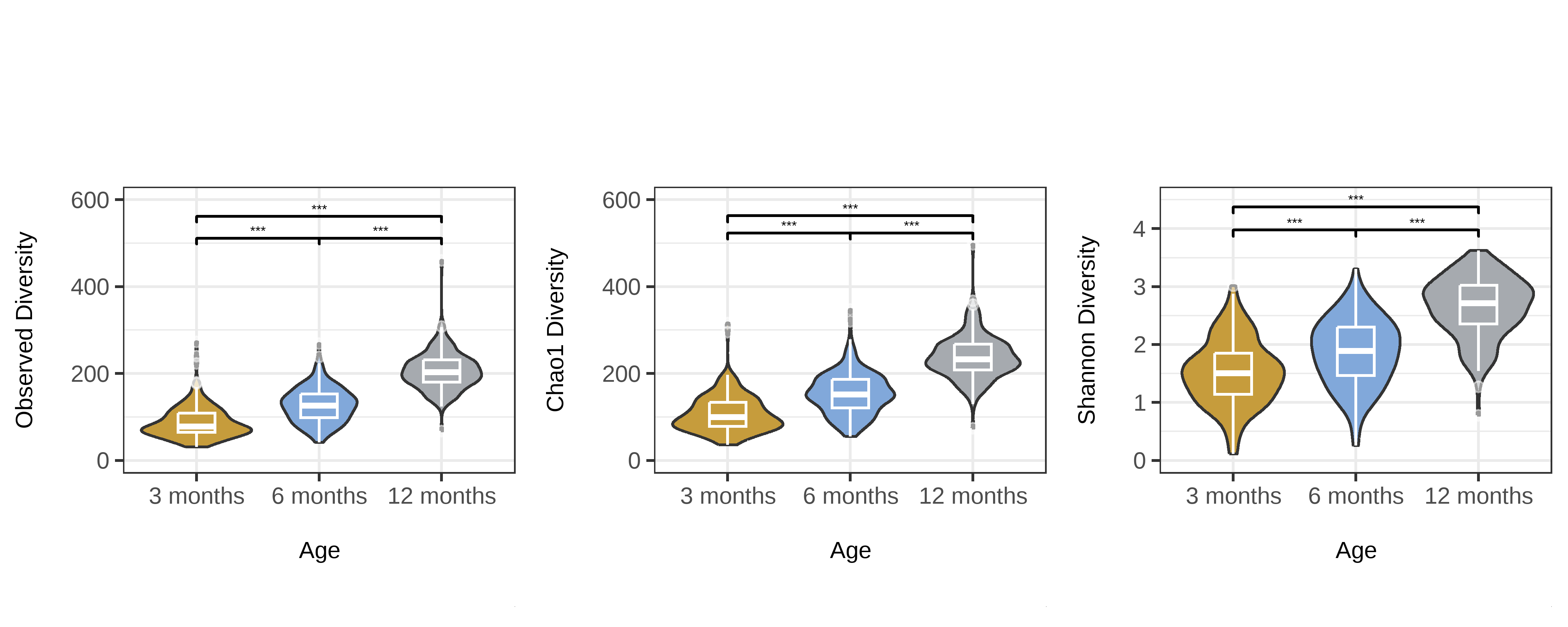

Supplement: S3 Fig — (TIF) [file pone.0291441.s004.tif]
