# Supplementary material for: Not simply a matter of parents—Infants’ sleep-wake patterns are associated with their regularity of eating
Source: PLoS One. 2023 Oct 5;18(10):e0291441. doi: 10.1371/journal.pone.0291441 (PMC10553286; doi:10.1371/journal.pone.0291441)
Supplement: S1 Table — Results of multilevel models and general linear models with the sleep composites as dependent variable and the Eating Regularity Index as an independent variable. (DOCX) [file pone.0291441.s005.docx]

**S1 Table. Sleep composites association with Eating Regularity Index**

Results of multilevel models and general linear models with the sleep composites as dependent variable and the Eating Regularity Index as an independent variable

|  | **Overall** |  | **3 months** |  | **6 months** |  | **12 months** |  |
| --- | --- | --- | --- | --- | --- | --- | --- | --- |
| **Sleep Activity** | **Estimate ± SEM** | **P value** | **Estimate ± SEM** | **P value** | **Estimate ± SEM** | **P value** | **Estimate ± SEM** | **P value** |
| Intercept | 1.739 ± 0.594 | 0.004 | 1.818 ± 1.598 | 0.257 | -0.879 ± 1.689 | 0.604 | 6.874 ± 3.359 | 0.043 |
| Eating Regularity Index | -1.516 ± 0.471 | 0.001 | -1.077 ± 1.361 | 0.430 | -1.859 ± 0.761 | 0.016 | -1.702 ± 0.592 | 0.005 |
| Ratio of meals from 7am to 7pm | -0.642 ± 0.505 | 0.205 | -1.763 ± 1.333 | 0.189 | -0.358 ± 0.809 | 0.659 | -0.689 ± 0.704 | 0.330 |
| Exact Age | -0.123 ± 0.014 | >0.001 | 0.003 ± 0.430 | 0.995 | 0.182 ± 0.233 | 0.436 | -0.519 ± 0.283 | 0.069 |
| Sex | -0.239 ± 0.091 | 0.009 | -0.181 ± 0.163 | 0.269 | -0.426 ± 0.137 | 0.002 | -0.111 ± 0.123 | 0.371 |
| BreastFeeding | 0.091 ± 0.133 | 0.493 | - | - | -0.119 ± 0.307 | 0.700 | 0.177 ± 0.144 | 0.223 |
| Meals number | 0.051 ± 0.031 | 0.098 | 0.044 ± 0.070 | 0.534 | 0.086 ± 0.047 | 0.069 | 0.059 ± 0.046 | 0.199 |
| BCQ Structure | 0.004 ± 0.135 | 0.979 | 0.077 ± 0.241 | 0.749 | 0.273 ± 0.213 | 0.202 | -0.148 ± 0.203 | 0.468 |
|  |  |  |  |  |  |  |  |  |
|  | **Overall** |  | **3 months** |  | **6 months** |  | **12 months** |  |
| **Sleep Timing** | **Estimate ± SEM** | **P value** | **Estimate ± SEM** | **P value** | **Estimate ± SEM** | **P value** | **Estimate ± SEM** | **P value** |
| Intercept | 3.468 ± 0.638 | >0.001 | 4.837 ± 1.814 | 0.009 | 6.275 ± 1.766 | 0.001 | 4.239 ± 3.487 | 0.226 |
| Eating Regularity Index | -1.211 ± 0.456 | 0.008 | -2.261 ± 1.533 | 0.143 | -1.537 ± 0.793 | 0.055 | -1.371 ± 0.619 | 0.029 |
| Ratio of meals from 7am to 7pm | 0.152 ± 0.486 | 0.755 | 0.231 ± 1.500 | 0.878 | -1.298 ± 0.843 | 0.126 | -1.254 ± 0.736 | 0.091 |
| Exact Age | -0.066 ± 0.013 | >0.001 | -0.544 ± 0.488 | 0.267 | -0.344 ± 0.245 | 0.163 | -0.069 ± 0.295 | 0.815 |
| Sex | 0.078 ± 0.126 | 0.533 | 0.133 ± 0.184 | 0.469 | 0.118 ± 0.143 | 0.412 | 0.075 ± 0.128 | 0.560 |
| BreastFeeding | -0.065 ± 0.119 | 0.587 | 0.044 ± 0.079 | 0.582 | -0.019 ± 0.307 | 0.950 | 0.398 ± 0.150 | 0.009 |
| Meals number | -0.006 ± 0.032 | 0.849 | 0.044 ± 0.079 | 0.582 | -0.017 ± 0.049 | 0.723 | -0.110 ± 0.048 | 0.023 |
| BCQ Structure | -0.843 ± 0.153 | >0.001 | -0.765 ± 0.272 | 0.006 | -0.973 ± 0.223 | >0.001 | -0.541 ± 0.211 | 0.012 |
|  |  |  |  |  |  |  |  |  |
|  | **Overall** |  | **3 months** |  | **6 months** |  | **12 months** |  |
| **Sleep Variability** | **Estimate ± SEM** | **P value** | **Estimate ± SEM** | **P value** | **Estimate ± SEM** | **P value** | **Estimate ± SEM** | **P value** |
| Intercept | 2.007 ± 0.671 | 0.003 | 1.969 ± 1.727 | 0.257 | 1.188 ± 1.615 | 0.463 | 8.677 ± 4.499 | 0.056 |
| Eating Regularity Index | -3.540 ± 0.529 | >0.001 | -4.767 ± 1.468 | 0.002 | -3.835 ± 0.735 | >0.001 | -3.635 ± 0.786 | >0.001 |
| Ratio of meals from 7am to 7pm | 0.700 ± 0.570 | 0.220 | 1.110 ± 1.443 | 0.443 | -0.029 ± 0.777 | 0.970 | 0.566 ± 0.940 | 0.548 |
| Exact Age | -0.044 ± 0.016 | 0.006 | -0.356 ± 0.466 | 0.447 | 0.202 ± 0.225 | 0.372 | -0.685 ± 0.377 | 0.072 |
| Sex | -0.123 ± 0.104 | 0.238 | -0.043 ± 0.175 | 0.808 | -0.216 ± 0.132 | 0.105 | 0.010 ± 0.164 | 0.952 |
| BreastFeeding | -0.062 ± 0.146 | 0.669 | - | - | -0.287 ± 0.279 | 0.305 | 0.273 ± 0.190 | 0.154 |
| Meals number | 0.107 ± 0.035 | 0.002 | 0.234 ± 0.076 | 0.003 | 0.091 ± 0.045 | 0.046 | 0.060 ± 0.061 | 0.323 |
| BCQ Structure | -0.297 ± 0.155 | 0.056 | -0.142 ± 0.261 | 0.587 | -0.252 ± 0.209 | 0.229 | 0.111 ± 0.271 | 0.682 |
|  |  |  |  |  |  |  |  |  |
|  | **Overall** |  | **3 months** |  | **6 months** |  | **12 months** |  |
| **Sleep Night** | **Estimate ± SEM** | **P value** | **Estimate ± SEM** | **P value** | **Estimate ± SEM** | **P value** | **Estimate ± SEM** | **P value** |
| Intercept | -2.830 ± 0.689 | >0.001 | -4.703 ± 1.978 | 0.019 | -3.611 ± 2.001 | 0.073 | -3.042 ± 3.956 | 0.443 |
| Eating Regularity Index | -0.257 ± 0.493 | 0.603 | -1.942 ± 1.680 | 0.250 | -0.895 ± 0.895 | 0.319 | 0.428 ± 0.698 | 0.540 |
| Ratio of meals from 7am to 7pm | 1.662 ± 0.537 | 0.002 | 4.042 ± 1.644 | 0.015 | 1.738 ± 0.952 | 0.070 | 2.799 ± 0.835 | 0.001 |
| Exact Age | 0.016 ± 0.016 | 0.328 | 0.060 ± 0.533 | 0.910 | 0.042 ± 0.276 | 0.880 | 0.000 ± 0.333 | 0.999 |
| Sex | -0.019 ± 0.119 | 0.873 | 0.111 ± 0.201 | 0.580 | -0.054 ± 0.162 | 0.738 | -0.061 ± 0.145 | 0.675 |
| BreastFeeding | -0.011 ± 0.128 | 0.930 | - | - | -0.197 ± 0.348 | 0.573 | -0.076 ± 0.172 | 0.657 |
| Meals number | 0.059 ± 0.035 | 0.090 | 0.074 ± 0.087 | 0.394 | 0.069 ± 0.055 | 0.213 | 0.059 ± 0.054 | 0.275 |
| BCQ Structure | 0.502 ± 0.162 | 0.002 | 0.819 ± 0.297 | 0.007 | 0.898 ± 0.252 | 0.001 | 0.242 ± 0.240 | 0.315 |
|  |  |  |  |  |  |  |  |  |
|  |  |  |  |  |  |  |  |  |
|  | **Overall** |  | **3 months** |  | **6 months** |  | **12 months** |  |
| **Sleep Day** | **Estimate ± SEM** | **P value** | **Estimate ± SEM** | **P value** | **Estimate ± SEM** | **P value** | **Estimate ± SEM** | **P value** |
| Intercept | 3.059 ± 0.447 | >0.001 | 6.027 ± 1.028 | >0.001 | 2.429 ± 1.090 | 0.028 | 3.386 ± 2.942 | 0.252 |
| Eating Regularity Index | 0.100 ± 0.360 | 0.781 | -0.390 ± 0.863 | 0.652 | 0.736 ± 0.489 | 0.135 | -0.162 ± 0.515 | 0.753 |
| Ratio of meals from 7am to 7pm | -0.889 ± 0.392 | 0.024 | -2.370 ± 0.845 | 0.006 | -0.655 ± 0.525 | 0.215 | -1.546 ± 0.623 | 0.014 |
| Exact Age | -0.219 ± 0.011 | >0.001 | -1.118 ± 0.278 | >0.001 | -0.282 ± 0.150 | 0.063 | -0.169 ± 0.247 | 0.496 |
| Sex | -0.037 ± 0.066 | 0.571 | 0.087 ± 0.104 | 0.404 | -0.025 ± 0.089 | 0.776 | -0.076 ± 0.108 | 0.480 |
| BreastFeeding | -0.199 ± 0.105 | 0.059 | - | - | -0.060 ± 0.182 | 0.742 | -0.124 ± 0.128 | 0.336 |
| Meals number | -0.002 ± 0.023 | 0.922 | 0.007 ± 0.045 | 0.872 | -0.012 ± 0.030 | 0.682 | -0.001 ± 0.041 | 0.983 |
| BCQ Structure | -0.314 ± 0.100 | 0.002 | -0.107 ± 0.154 | 0.487 | -0.260 ± 0.138 | 0.062 | -0.385 ± 0.179 | 0.034 |
