# Supplementary material for: Not simply a matter of parents—Infants’ sleep-wake patterns are associated with their regularity of eating
Source: PLoS One. 2023 Oct 5;18(10):e0291441. doi: 10.1371/journal.pone.0291441 (PMC10553286; doi:10.1371/journal.pone.0291441)
Supplement: S2 Table — Results of multilevel models and general linear models with the different Phyla as dependent variable and the Eating Regularity Index as independent variable. (DOCX) [file pone.0291441.s006.docx]

**S2 Table. Phyla association with Eating Regularity Index**

Results of multilevel models and general linear models with the different Phyla as dependent variable and the Eating Regularity Index as independent variable

| **Firmicutes** | Estimate ± SEM | P value |  | **Bacteroidetes** | Estimate ± SEM | P value |
| --- | --- | --- | --- | --- | --- | --- |
| Intercept | -0.002 ± 0.145 | 0.989 |  | Intercept | 0.107 ± 0.189 | 0.572 |
| Eating Regularity Index | 0.389 ± 0.115 | 0.001 |  | Eating Regularity Index | -0.125 ± 0.149 | 0.400 |
| Ratio of meals from 7am to 7pm | 0.049 ± 0.124 | 0.696 |  | Ratio of meals from 7am to 7pm | 0.217 ± 0.161 | 0.177 |
| Exact Age | 0.030 ± 0.003 | 0.000 |  | Exact Age | 0.005 ± 0.004 | 0.217 |
| Sex | -0.045 ± 0.023 | 0.053 |  | Sex | -0.013 ± 0.031 | 0.671 |
| BreastFeeding | -0.051 ± 0.032 | 0.110 |  | BreastFeeding | 0.006 ± 0.041 | 0.887 |
| Meals number | -0.021 ± 0.008 | 0.005 |  | Meals number | -0.001 ± 0.010 | 0.900 |
| BCQ Structure | 0.005 ± 0.033 | 0.891 |  | BCQ Structure | 0.018 ± 0.044 | 0.679 |
|  |  |  |  |  |  |  |
| **Proteobacteria** | Estimate ± SEM | P value |  | **Actinobacteria** | Estimate ± SEM | P value |
| Intercept | 0.154 ± 0.103 | 0.138 |  | Intercept | 0.720 ± 0.196 | 0.000 |
| Eating Regularity Index | 0.025 ± 0.084 | 0.765 |  | Eating Regularity Index | -0.237 ± 0.158 | 0.133 |
| Ratio of meals from 7am to 7pm | -0.032 ± 0.091 | 0.729 |  | Ratio of meals from 7am to 7pm | -0.217 ± 0.170 | 0.203 |
| Exact Age | -0.014 ± 0.003 | 0.000 |  | Exact Age | -0.024 ± 0.005 | 0.000 |
| Sex | 0.008 ± 0.015 | 0.607 |  | Sex | 0.044 ± 0.031 | 0.150 |
| BreastFeeding | 0.025 ± 0.025 | 0.311 |  | BreastFeeding | 0.024 ± 0.043 | 0.583 |
| Meals number | -0.001 ± 0.005 | 0.919 |  | Meals number | 0.020 ± 0.010 | 0.052 |
| BCQ Structure | 0.016 ± 0.023 | 0.501 |  | BCQ Structure | -0.033 ± 0.045 | 0.461 |
|  |  |  |  |  |  |  |
| **Verrucomicrobia** | Estimate ± SEM | P value |  | **Fusobacteria** | Estimate ± SEM | P value |
| Intercept | 0.032 ± 0.025 | 0.192 |  | Intercept | -6.96e-04 ± 2.99e-04 | 0.020 |
| Eating Regularity Index | -0.022 ± 0.021 | 0.288 |  | Eating Regularity Index | -2.77e-04 ± 2.49e-04 | 0.267 |
| Ratio of meals from 7am to 7pm | -0.012 ± 0.022 | 0.603 |  | Ratio of meals from 7am to 7pm | 6.51e-04 ± 2.69e-04 | 0.016 |
| Exact Age | 0.001 ± 0.001 | 0.041 |  | Exact Age | 1.74e-05 ± 7.93e-06 | 0.029 |
| Sex | 0.001 ± 0.004 | 0.852 |  | Sex | 1.74e-05 ± 4.32e-05 | 0.687 |
| BreastFeeding | -0.012 ± 0.006 | 0.037 |  | BreastFeeding | 6.33e-05 ± 7.00e-05 | 0.366 |
| Meals number | 0.000 ± 0.001 | 0.891 |  | Meals number | 4.20e-05 ± 1.55e-05 | 0.007 |
| BCQ Structure | -0.002 ± 0.006 | 0.683 |  | BCQ Structure | 2.07e-05 ± 6.72e-05 | 0.758 |
|  |  |  |  |  |  |  |
| **Tenericutes** | Estimate ± SEM | P value |  | **Chloroflexi** | Estimate ± SEM | P value |
| Intercept | 2.20e-06 ± 1.52e-06 | 0.068 |  | Intercept | 1.48e-03 ± 2.09e-02 | 0.149 |
| Eating Regularity Index | -5.45e-07 ± 1.27e-06 | 0.973 |  | Eating Regularity Index | -4.12e-03 ± 1.74e-02 | 0.667 |
| Ratio of meals from 7am to 7pm | 1.29e-07 ± 1.37e-06 | 0.275 |  | Ratio of meals from 7am to 7pm | -1.56e-02 ± 1.88e-02 | 0.925 |
| Exact Age | -3.34e-08 ± 4.01e-08 | 0.502 |  | Exact Age | 1.58e-03 ± 5.51e-04 | 0.405 |
| Sex | 1.22e-07 ± 2.20e-07 | 0.387 |  | Sex | 2.85e-03 ± 3.03e-03 | 0.582 |
| BreastFeeding | -2.41e-07 ± 3.55e-07 | 0.849 |  | BreastFeeding | 6.73e-03 ± 4.87e-03 | 0.497 |
| Meals number | -5.52e-08 ± 7.91e-08 | 0.241 |  | Meals number | 9.12e-04 ± 1.09e-03 | 0.486 |
| BCQ Structure | -3.81e-07 ± 3.42e-07 | 0.120 |  | BCQ Structure | -3.78e-03 ± 4.71e-03 | 0.267 |
|  |  |  |  |  |  |  |
| **Cyanobacteria** | Estimate ± SEM | P value |  | **Deferribacteres** | Estimate ± SEM | P value |
| Intercept | 0.001 ± 0.021 | 0.943 |  | Intercept | 1.48e-03 ± 2.09e-02 | 0.531 |
| Eating Regularity Index | -0.004 ± 0.017 | 0.813 |  | Eating Regularity Index | -4.12e-03 ± 1.74e-02 | 0.194 |
| Ratio of meals from 7am to 7pm | -0.016 ± 0.019 | 0.408 |  | Ratio of meals from 7am to 7pm | -1.56e-02 ± 1.88e-02 | 0.929 |
| Exact Age | 0.002 ± 0.001 | 0.004 |  | Exact Age | 1.58e-03 ± 5.51e-04 | 0.388 |
| Sex | 0.003 ± 0.003 | 0.348 |  | Sex | 2.85e-03 ± 3.03e-03 | 0.109 |
| BreastFeeding | 0.007 ± 0.005 | 0.168 |  | BreastFeeding | 6.73e-03 ± 4.87e-03 | 0.071 |
| Meals number | 0.001 ± 0.001 | 0.402 |  | Meals number | 9.12e-04 ± 1.09e-03 | 0.124 |
| BCQ Structure | -0.004 ± 0.005 | 0.423 |  | BCQ Structure | -3.78e-03 ± 4.71e-03 | 0.981 |
|  |  |  |  |  |  |  |
| **TM7** | Estimate ± SEM | P value |  |  |  |  |
| Intercept | -2.42e-05 ± 1.25e-04 | 0.847 |  |  |  |  |
| Eating Regularity Index | -3.45e-05 ± 1.04e-04 | 0.740 |  |  |  |  |
| Ratio of meals from 7am to 7pm | -5.77e-05 ± 1.12e-04 | 0.606 |  |  |  |  |
| Exact Age | 6.92e-06 ± 3.23e-06 | 0.033 |  |  |  |  |
| Sex | 4.02e-05 ± 1.86e-05 | 0.031 |  |  |  |  |
| BreastFeeding | 1.30e-05 ± 2.88e-05 | 0.651 |  |  |  |  |
| Meals number | 4.40e-06 ± 6.54e-06 | 0.501 |  |  |  |  |
| BCQ Structure | 7.62e-06 ± 2.85e-05 | 0.789 |  |  |  |  |
