# Supplementary material for: Not simply a matter of parents—Infants’ sleep-wake patterns are associated with their regularity of eating
Source: PLoS One. 2023 Oct 5;18(10):e0291441. doi: 10.1371/journal.pone.0291441 (PMC10553286; doi:10.1371/journal.pone.0291441)
Supplement: Attachment — Submitted filename: Revisions_PointByPoint_230712.pdf [file pone.0291441.s007.pdf]

Dear Prof. Nakamura,

We thank the editor and the reviewers for their positive feedback on our manuscript PONE-D-23-13344 entitled "Not simply a matter of parents - Infants' sleep-wake patterns are associated with their regularity of eating" and for considering a revised version for publication in PLOS ONE.

We have thoroughly revised the entire manuscript taking into consideration the comments and suggestions raised by the reviewers. We highlight these changes in the manuscript in yellow. We are grateful that the quality of the manuscript has considerably improved through this feedback. Please find our point-to-point answers to the comments and questions below.

Point-by-point response

Reviewer #1:

"*My major concern is the that the "composite" of the sleep wake patterns were created with a mixed of factors that could be controversial. As an example Sleep activity: refers to sleep efficiency (that was not defined), sleep movements (other unit of measure) and nocturnal wake episodes. I saw similar confusion in the other 3 variables which conformed the index. Nevertheless the results were in an appropriate direction*."

We acknowledge the concerns raised by the reviewer regarding the utilization of sleep composites and the eating regularity index (ERI), which may be subject to controversy. We conducted a careful selection and computation of the variables, considering their inherent meanings. In a previous study (Schoch et al., 2020, Sensors), we derived five sleep composites from 48 commonly-used sleep variables: Sleep Night, Sleep Day, Sleep Activity, Sleep Timing, and Sleep Variability. Unlike the numerous individual variables commonly employed, these sleep composites provide a more representative means of capturing sleep patterns and are associated with behavioral development, making them more reliable variables for research purposes. The sleep composites accurately reflect established sleep dynamics throughout infancy. For instance, Sleep Day represents naps, Sleep Activity reflects sleep efficiency and consolidation, Sleep Variability indicates day-to-day stability, and Sleep Night represents nighttime sleep, which slightly increases with age, while Sleep Timing becomes earlier. Consequently, we included the sleep composites of relevance for the research question in our analysis and also incorporated a simplified metric for eating regularity, known as the Eating Regularity Index (ERI). By utilizing these composites and the ERI, we significantly reduced the number of sleep variables, thereby minimizing the risk of type I errors. Additionally, as the first study to employ the Eating Regularity Index, our objective was to comprehensively explore the associations between eating regularity and sleep.

We adapted these clarifications to the manuscript (lines 108-112).

“***The authors pointed out the interaction of the eating behavior over sleep, but they did not analyze the possibility that sleep patterns could be the predictor factor, or at least mention that they might be interrelated*.**”
The reviewer raises a highly interesting point, which will be very relevant to examine in the near future. We would like to acknowledge the possibility that sleep patterns could also be driving eating behaviors - so it is indeed plausible that this relationship is bidirectional. We have now computed a multiple regression with ERI as the dependent variable and the five sleep composites as the predictor, which reveals that the reported associations between the ERI and sleep composites (Sleep Activity, Sleep Timing and Sleep Variability) are significant in both directions (see table below). We prefer to abstain from presenting this table directly in the manuscript, but could leave this decision to the editor.

	**Coefficients**	**Standard Error**	**t stat**	**df**	**p value**
**Intercept**	0,5424	0,0057	94,3505	437,7858	<0,000001
**Sleep Variability**	-0,0226	0,0057	-3,9666	390,8267	0,000087
**Sleep Timing**	-0,0139	0,0060	-2,2955	403,8669	0,022219
**Sleep Day**	-0,0045	0,0056	-0,7969	392,3985	0,425994
**Sleep Night**	-0,0038	0,0052	-0,7411	407,6902	0,459066
**Sleep Activity**	-0,0163	0,0056	-2,9006	396,5213	0,003932

Multiple regression model with the five sleep composites as predictors and the ERI as dependent variable

We have now clarified the point of possible bidirectionality in the discussion section (lines 368-376).

Reviewer #2:

“***The original research is novel and valuable addition to the existing literature. The eating patterns are set very early in life and are getting correlated to sleep patterns is a valuable finding. Parental efforts to regularise it early is also rewarding. The attempt to link it with Gut Microbiota is noteworthy. The paper does not capture the breastfeeding pattern, weaning foods, its variety as all these factors can also influence eating, sleep pattern and gut microbiota.”***
We appreciate this positive feedback and agree that the breastfeeding pattern and weaning food might have an impact on the development of eating behavior and sleeping patterns. We included for every assessment timepoint the indication of whether the participant was breastfed or not (line 165, 167; control variable: breastfeeding 0 for not or rarely breastfed, 1 for occasionally, regularly, or daily breastfeeding), which captures the weaning time in some rough form. We believe that the reviewer’s comment on the timepoint of weaning and transitions of food is highly relevant and we are indeed taking next steps in a new investigation to examine

how the different types of food or milk in conjunction with the time at which they are consumed can impact the association between nutrition and sleep in infancy.

Additional changes

We have now switched the axes in Figure 2 (line 232) to align with Figure 3 (line 303) and make it more comprehensible.

Journal requests

1. **We have adjusted the formatting to fit the journal's needs.**

2. Funding: 2. We note that the grant information you provided in the 'Funding Information' and 'Financial Disclosure' sections do not match.
   When you resubmit, please ensure that you provide the correct grant numbers for the awards you received for your study in the 'Funding Information' section.
   We have made a new document of funding documentation to be insterted to the article

3. Competing interest statement
   We have adapted this point as requested and uploaded it as a separate document.

4. Data availability
   Ethical restrictions (cantonal ethics committee of Zurich, Switzerland) apply to this paper, which prevents the public sharing of individual data that contain potentially sensitive information. Participants giving consent to the study were not informed that their and their children's data, might be shared publicly. Anonymized data are available from the corresponding author on reasonable request and pending ethics approval.

5. **Reference to Figure 3 was inserted.**

6. **Captions for your Supporting Information files were inserted at the end of the manuscript**
